# Bermudagrass Management in the Southern Piedmont U.S. IV. Soil Surface Nitrogen Pools

**DOI:** 10.1100/tsw.2001.89

**Published:** 2001-09-24

**Authors:** Alan J. Franzluebbers, John A. Stuedemann

**Affiliations:** U.S Department of Agriculture-Agricultural Research Service, Natural Resource Conservation Center, Watkinsville, GA 30677-2373, USA

## Abstract

The fate of nitrogen (N) applied in forage-based agricultural systems is important for understanding the long-term production and environmental impacts of a particular management strategy. We evaluated the factorial combination of three types of N fertilization (inorganic, crimson clover [Trifolium incarnatum L.] cover crop plus inorganic, and chicken [Gallus gallus] broiler litter pressure and four types of harvest strategy (unharvested forage, low and high cattle [Bos Taurus] grazing pressure, and monthly haying in summer) on surface residue and soil N pools during the first 5 years of ‘Coastal’ bermudagrass (Cynodon dactylon [L.] Pers.) management. The type of N fertilization used resulted in small changes in soil N pools, except at a depth of 0 to 2 cm, where total soil N was sequestered at a rate 0.2 g ‧ kg‧ year1 greater with inorganic fertilization than with other fertilization strategies. We could account for more of the applied N under grazed systems (76–82%) than under ungrazed systems (35–71%). As a percentage of applied N, 32 and 48% were sequestered as total soil N at a depth of 0 to 6 cm when averaged across fertilization strategies under low and high grazing pressures, respectively, which was equivalent to 6.8 and 10.3 g ‧ m ‧ year. Sequestration rates of total soil N under the unharvested-forage and haying strategies were negligible. Most of the increase in total soil N was at a depth of 0 to 2 cm and was due to changes in the particulate organic N (PON) pool. The greater cycling of applied N into the soil organic N pool with grazed compared with ungrazed systems suggests an increase in the long-term fertility of soil.

## INTRODUCTION

Nitrogen is an essential nutrient for developing and maintaining the productive capacity of grass-management systems, especially on weathered soils of the warm, humid southeastern U.S. Bermudagrass hybrids are adapted to the conditions of the southeastern U.S., responding with dramatic increases in biomass production to applied N at rates up to 50 g · m$^{-2}$ · year$^{-1}$ when hayed [1,2]. However, when cattle graze, most of the N accumulated in the forage and subsequently consumed by the cattle is redeposited to the soil via dung and urine[3].

The fate of recycled N in pasture systems is contingent on a number of environmental and biological factors and can therefore be influenced by the choice of management. Numerous transformations can contribute to the sequestration or loss of N from an ecosystem[4]. Sequestration of N is most notable via incorporation into organic matter, which can be labile or recalcitrant depending on its biochemical structure[5]. Losses of N can occur through ammonia volatilization, particularly from urine deposits[6]. Denitrification requires a readily oxidizable source of carbon, the presence of nitrate, and low availability of oxygen, all of which can occur under wet pasture conditions[7]. Although perennial grass systems tend to be more efficient at capturing inorganic N in the soil than annual crop systems because of their extensive root system, nitrate can also leach beyond the plant root zone if the applied N is excessive[8]. Similarly, grass systems tend to have higher aggregate stability and infiltration rates than tilled crop systems, but surface runoff of inorganic and organic N can occur with heavy rainfall[4].

The cycling of N from fertilizer to soil to forage to cattle to manure to soil is a biologically mediated process that transforms inorganic N to organic N via mineralization and immobilization. Degraded soils with low organic matter could be a significant sink for organic N when land is converted to improved grass-management systems. How harvest management and the source of nutrients affect the accumulation of soil organic N is not well defined. Our objective was to evaluate how types of fertilization and harvest strategy affected soil organic N accumulation and depth distribution during the first 5 years of 'Coastal' bermudagrass establishment on a previously degraded soil in the Southern Piedmont U.S.

## EXPERIMENTAL METHODS

### Site Characteristics

A 15-ha upland field (33°22′N, 83°24′W) near Farmington, GA had previously been conventionally cultivated with traditional annual grain and fiber crops for several decades prior to the grassland establishment by sprigging of 'Coastal' bermudagrass in 1991. Sampled on a 30-m grid, the frequency of soil series was 46% Madison, 22% Cecil, 13% Pacolet, 5% Appling, 2% Wedowee (fine, kaolinitic, thermic Typic Kanhapludults), 11% Grover (fine-loamy, micaceous, thermic Typic Hapludults), and 1% Louisa (loamy, micaceous, thermic, shallow Ruptic-Ultic Dystrudepts). Soil textural frequency of the Ap horizon was 75% sandy loam, 12% sandy clay loam, 8% loamy sand, and 4% loam. Depth of the Ap horizon was 21 ± 2 cm. Mean annual temperature is 16.5°C, rainfall is 1250 mm, potential evaporation is 1560 mm, and elevation is 205 to 215 m above mean sea level.

### Experimental Design

The experimental design was a randomized complete block with treatments in a split-plot arrangement in each of three blocks, which were delineated by landscape features (i.e., slight, moderate, and severe erosion classes). Main plots were fertilization strategy (n = 3) and split-plots were harvest strategy (n = 4), for a total of 36 experimental units. Grazed paddocks were 0.69 ± 0.03 ha. Each paddock contained a 3 · 4 m shaded area, a mineral feeder, and a water trough placed in a line 15 m long near the top of the landscape. Unharvested and hayed exclosures within each paddock were 100 $m^2$.

Fertilization strategy was ca. 20 g total N · $m^{-2}$ · $year^{-1}$ supplied (1) inorganically as $NH_4NO_3$ broadcast in split applications in May and July, (2) by crimson clover cover crop plus supplemental inorganic fertilizer with half of the N assumed fixed by clover biomass and the other half as $NH_4NO_3$ broadcast in July, and (3) by broiler litter broadcast in split applications in May and July. Details of annual N fertilizer applications are described in Table 1. Crimson clover was direct-drilled in clover treatments at 1 g · $m^{-2}$ in October each year. All paddocks were mowed in late April following soil sampling, and the residue was allowed to decompose (i.e., clover biomass in clover plus inorganic treatment and winter annual weeds in other treatments).

Harvest strategy mimicked a gradient in forage utilization consisting of (1) unharvested forage (biomass cut and left in place at the end of growing season), (2) low grazing pressure (put-and-take system to maintain a target of ca. 300 g · $m^{-2}$ of available forage), (3) high grazing pressure (put-and-take system to maintain a target of ca. 150 g · $m^{-2}$ of available forage), and (4) monthly haying in summer to remove aboveground biomass at a 4-cm height. Yearling Angus steers grazed paddocks during a 140-d period from mid-May until early October each year, except during the first year of treatment implementation (1994), when grazing began in July due to repairs to infrastructure following a tornado. No grazing occurred in the winter. Animals were weighed, available forage determined, and paddocks restocked on a monthly basis.

### Sampling and Analyses

Soil and surface residues were sampled in April prior to grazing. Hayed and unharvested exclosures were sampled in July rather than April during 1994. Sampling locations in grazed paddocks were within a 3-m radius of points on a 30-m grid. Due to the nonuniform dimensions of paddocks, sampling sites within a paddock varied from 4 to 9, averaging 7 ± 1. Each hayed and unharvested exclosure had 2 fixed sampling locations. Surface residue was collected from a 0.25-$m^2$ area at each sampling point following removal of vegetation at a height of ca. 4 cm. Surface residue, including plant stubble, was cut to the mineral surface

**TABLE 1**
**Rate of N Fertilization (g · $m^{-2}$ · $year^{-1}$)**

| Fertilization strategy | 1994 | 1995 | 1996 | 1997 | 1998 | 5-year mean |
|---|---|---|---|---|---|---|
| Inorganic | 21.1 | 20.2 | 25.0 | 23.8 | 22.4 | 22.5 |
| Clover + inorganic[a] | 21.1 | 10.1 | 13.2 | 12.0 | 11.1 | 13.5 |
| Broiler litter | 19.5 | 21.6 | 16.4 | 22.3 | 17.2 | 19.4 |

[a] An additional 11 g N · $m^{-2}$ · $year^{-1}$ was assumed to be supplied in clover cover crop biomass through biological N fixation from 1995 to 1998.

with battery-powered hand shears, bagged, and dried at 70°C for several days. During 1994 and 1995, soil was sampled at depths of 0 to 2, 2 to 4, and 4 to 6 cm from the composite of two 8.5-cm-diam cores within each sampling location. From 1996 to 1998, soil was sampled to the same depths from the composite of nine 4.1-cm-diameter cores within each sampling location. Soil was air-dried and ground to <2 mm in a mechanical grinder in 1994 and 1995. Soil was oven-dried (55°C, 72 h) and gently crushed to pass a 4.75-mm screen in all other years.

Beginning in February 1999, sampling strategy was changed to (1) more directly address the zonal changes of soil properties in response to animal behavior near shade and water sources and (2) collect soil to deeper depths. Surface residue was collected from a composite of eight 0.04-$m^2$ areas randomly selected within each of three zones within paddocks (i.e., 0 to 30, 30 to 70, and 70 to 120 m from livestock shades) and within each exclosure. Surface residue was processed as described previously. A single 4.1-cm-diameter soil core was collected from each of the eight residue sampling sites and composited. Soil was collected at depths of 0 to 3, 3 to 6, 6 to 12, and 12 to 20 cm, oven-dried (55°C, 72 h), and gently crushed to pass a 4.75-mm screen.

Soil bulk density was calculated from the oven-dried soil weight (55°C) and pooled-core volume (2.26–8.45 · $10^{-4}$ $m^3$, depending on depth of sampling). Surface residue was ground to <1 mm and a 20- to 30-g soil subsample from each composite sample was ground to a fine powder in a ball mill for 3 min prior to analysis of total N with dry combustion at 1350°C (Leco CNS-2000, St. Joseph, MI)[a]. Total soil organic C and S, extractable and total P, and bulk density were reported elsewhere[9,10].

Particulate organic matter was collected from a 20- to 55-g subsample (subsample weight inversely proportional to estimated soil organic matter content) that was shaken with 100 ml of 0.01 *M* $Na_4P_2O_7$ for 16 h on a reciprocating shaker and then passed through a 0.05-mm screen. Sand-size material not passing the screen was transferred to a drying bottle, dried at 55°C for 72 h, weighed, ground in a ball mill for 3 min, and analyzed for PON using dry combustion. Particulate organic C was reported elsewhere[11].

Potential N mineralization was determined by placing a 20- to 55-g subsample of soil that was packed to 1.1 to 1.3 Mg · $m^{-3}$ in 60-ml-graduated glass jars, wetting the subsample to 50% water-filled pore space, and placing it in a 1-l canning jar along with 10 ml of 1 *M* NaOH to trap $CO_2$ and a vial of water to maintain humidity. Subsample weight was inversely proportional to estimated soil organic matter content. Density of samples in glass jars was the same within a soil depth and year, but density was different among depths and years depending on the general quantity of organic matter. Samples were incubated at 25 ± 1°C for 24 d. Potential N mineralization was the difference in inorganic N concentration between 0 and 24 d of incubation. Inorganic N ($NH_4$–N + $NO_2$–N + $NO_3$–N) was determined from the filtered extract of a 10-g subsample of dried (55°C for 48 h) and sieved (<2 mm) soil that was shaken with 20 ml of 2 *M* KCl for 30 min by salicylate-nitroprusside and Cd-reduction autoanalyzer techniques[12]. Potential C mineralization was reported elsewhere[11].

Data from multiple samples within an experimental unit were averaged and not considered as a source of variation in the analysis of variance (see SAS Institute)[13]. Within-depth, across-depth, within-year, and across-year analyses were conducted according to the split-plot design with three blocks. Across-depth analyses accounted for differences in the bulk density of soil in calculating areal estimates of soil N pools to a depth of 6 cm. Across-year analyses considered years as repeated measures. Effects were considered significant at P = 0.1.

## RESULTS AND DISCUSSION

### Harvest Strategy Impacts

Total N concentration of soil was relatively uniformly distributed with soil depth at the beginning of the experiment in 1994, but became increasingly stratified with time under all management systems (Fig. 1). Harvest strategy had the most significant influence on total N accumulation with time, especially at a depth of 0 to 2 cm. At the end of 4 years of management, total N concentration at a depth of 0 to 2 cm was significantly different among all harvest strategies, averaging 4.2, 3.5, 2.5, and 1.9 g · $kg^{-1}$ under high grazing pressure, low grazing pressure, unharvested-forage, and haying strategies, respectively. Total N concentration was also greater under both grazing pressures than under the unharvested-forage or haying strategies at depths of 2 to 4 and 4 to 6 cm, although the magnitude of difference was reduced to 0.2 and 0.1 g · $kg^{-1}$, respectively.

Total N concentration of soil increased significantly with time under all harvest strategies at a depth of 0 to 2 cm, but only under low and high cattle-grazing pressures at a depth of 2 to 4 cm (Table 2). At a depth of 4 to 6 cm, total N concentration did not change with time under cattle grazing and decreased significantly with time under unharvested-forage and haying strategies. Total N concentration of the 0- to 2-cm depth of soil quadrupled during 4 years under high grazing pressure, whereas it only doubled under haying.

Cattle-grazing systems led to significantly greater total N concentration of soil than unharvested forage at all soil depths (Fig. 1; Table 2). Total N accumulation was 20% greater with high than with low grazing pressure at a depth of 0 to 2 cm, but similar between grazing pressures at other soil depths. Total N accumulation was greater with unharvested forage than with haying at a depth of 0 to 2 cm, but similar between these strategies at other soil depths. On an areal basis to a depth of 6 cm, total N increased significantly during 4 years with low and high grazing pressures, but remained unchanged with unharvested forage and haying (Table 3). The rate of total N accumulation under high grazing pressure was ca. 50% greater than under low grazing pressure. These results suggest that cattle grazing returns a large portion of the consumed N in forage back to the soil as organically bound N.

Concentration of PON was slightly stratified at the beginning of the experiment, but became much more stratified with time under all harvest strategies (Fig. 1). Changes in PON

[a] Trade and company names are included for the benefit of the reader and do not imply any endorsement or preferential treatment of the product listed by the U.S. Department of Agriculture.

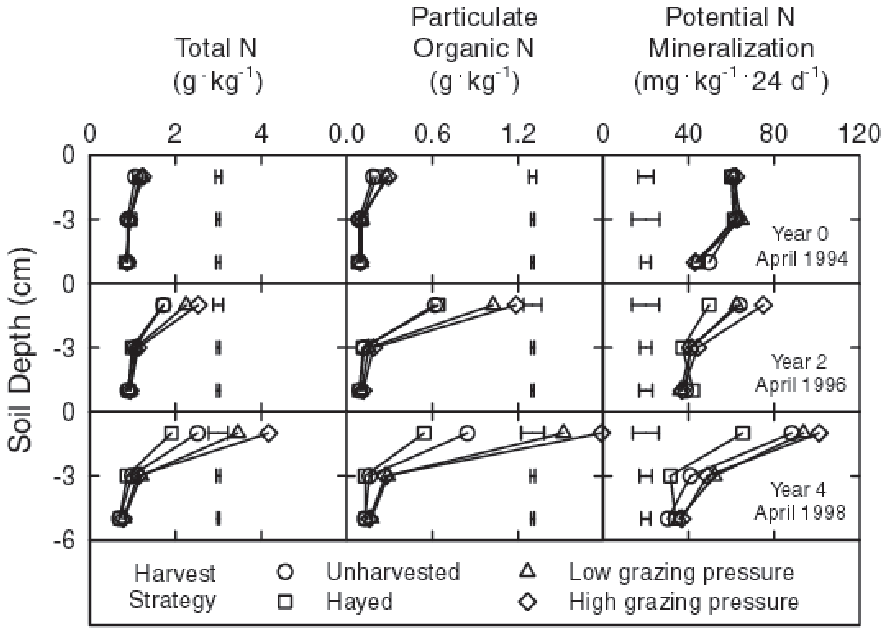

**FIGURE 1.** Depth distribution of total N, particulate organic N, and potential N mineralization during 24 d as affected by harvest strategy at the end of 0, 2, and 4 years of forage management. Error bars indicate LSD at P = 0.1 to separate means of harvest strategy within a soil depth.

mirrored those of total N, although harvest strategy impacts became more pronounced with this passive pool of organic N. PON increased with time under all harvest strategies at all soil depths, although the rate of increase was greatest at a depth of 0 to 2 cm (Table 2). Cattle grazing resulted in greater increases in PON than did unharvested forage or haying at all soil depths. On an areal basis to a depth of 6 cm, PON accumulation was greater under high than under low grazing pressure; PON accumulations under both were 2.5 to 4.3 times greater than under unharvested-forage or haying strategies (Table 3).

The rate of change in non-PON was greater under cattle grazing than under unharvested forage or haying at a depth of 0 to 2 cm, but not consistently at lower soil depths (Table 2). Non-PON accumulated at a depth of 0 to 2 cm, remained unchanged with time at a depth of 2 to 4 cm, and decreased with time at a depth of 4 to 6 cm. It appears that an increase in PON at a depth of 2 to 6 cm, due to root development with increasing pasture maturity, is important to offset the loss of non-PON resulting from steady-state decomposition of soil organic matter. On an areal basis to a depth of 6 cm, non-PON increased with time only under high grazing pressure, remained unchanged with time under low grazing pressure, and decreased with time under unharvested-forage and haying strategies (Table 3). The non-PON pool is assumed to represent a stable fraction of organic matter with a relatively slow turnover time.

Potential N mineralization was relatively uniform with soil depth at the beginning of the experiment, but also became increasingly stratified with depth during 4 years of management, much like total N and PON (Fig. 1). However, potential N mineralization, as a measure of the active organic N, behaved somewhat differently than the passive PON and total N pools. At a depth of 0 to 2 cm, potential N mineralization increased significantly with time under both cattle-grazing strategies, as well as when unharvested, but not when hayed (Table 2). At a depth of 2 to 4 cm, potential N mineralization decreased with time under all harvest strategies, although more under nongrazing than under grazing strategies. On an areal basis to a depth of 6 cm, potential N mineralization decreased significantly with time under all harvest strategies, which was in contrast to the positive temporal trends in total N and PON (Table 3). Also in contrast to other organic N pools, the temporal change in potential N mineralization under the unharvested-forage strategy was not significantly different from those under low and high grazing pressures.

Changes in the quality of soil organic N pools during 4 years of harvest management were evaluated from the ratios of particulate and mineralizable pools to total N and as C to N ratios of various pools (Table 4). PON as a proportion of total N increased with time under all harvest strategies at all soil depths. Cattle-grazing strategies led to a greater enrichment of soil with PON with time than did unharvested-forage or haying strategies at all soil depths. At the beginning of the experiment, PON at a depth of 0 to 6 cm averaged 16% of total N, but increased to an average of 39% of total N at the end of 4 years of cattle grazing and to 26% of total N at the end of 4 years of unharvested-forage or haying strategies. The increase in the proportion of total N as PON could be viewed as an improvement in soil quality that could lead to more efficient nutrient cycling. A significant decrease in the particulate organic C to N ratio occurred with time under cattle-grazing strategies, but no change in this ratio occurred with time under unharvested-forage or haying strategies. This result suggests that the greater total N accumulated under cattle grazing was also of higher quality (i.e., with a lower C to N ratio) than when ungrazed. The greater proportion of total N as PON and the lower C to N ratio of particulate organic matter under cattle grazing should improve nutrient cycling, as

**TABLE 2**
**Rate of Change in N Pools, Based on Linear Regression, During the First 4 Years of Management as a Function of Soil Depth and in Response to Harvest Strategy Averaged across Fertilization Strategies**

| Soil depth (cm) | Intercept | Harvest strategy UH | LG | HG | H | $LSD_{(P=0.1)}$ |
|---|---|---|---|---|---|---|
| | $g \cdot kg^{-1}$ | $g \cdot kg^{-1} \cdot year^{-1}$ | | | | |
| **Total N (TN)** | | | | | | |
| 0 to 2 | 1.04 | 0.36 | 0.64 | 0.77 | 0.27 | 0.08 |
| 2 to 4 | 0.95 | 0.01 | 0.05 | 0.05 | –0.01 | 0.02 |
| 4 to 6 | 0.94 | –0.05 | –0.03 | –0.01 | –0.05 | 0.03 |
| **Particulate organic N (PON)** | | | | | | |
| 0 to 2 | 0.31 | 0.15 | 0.35 | 0.41 | 0.09 | 0.05 |
| 2 to 4 | 0.10 | 0.02 | 0.05 | 0.05 | 0.02 | 0.01 |
| 4 to 6 | 0.09 | 0.01 | 0.03 | 0.02 | 0.01 | 0.01 |
| **Non-particulate organic N (Non-PON)** | | | | | | |
| 0 to 2 | 0.86 | 0.18 | 0.25 | 0.33 | 0.14 | 0.06 |
| 2 to 4 | 0.84 | –0.01 | 0.00 | 0.00 | –0.03 | 0.02 |
| 4 to 6 | 0.82 | –0.06 | –0.05 | –0.03 | –0.06 | 0.03 |
| | $mg \cdot kg^{-1}$ | $mg \cdot kg^{-1} \cdot year^{-1}$ | | | | |
| **Potential N Mineralization during 24 d (NMIN)** | | | | | | |
| 0 to 2 | 57.8 | 6.7 | 9.7 | 11.3 | 2.5 | 4.2 |
| 2 to 4 | 58.3 | –5.9 | –3.8 | –4.3 | –8.0 | 2.2 |
| 4 to 6 | 45.5 | –2.1 | –1.7 | –1.6 | –1.5 | 1.9 |

*Note:* UH is unharvested, LG is low grazing pressure, HG is high grazing pressure, and H is hayed. Linear regression was of the form, (N pool = $\beta_0 + \beta_1 \cdot$ years), where $\beta_0$ is the intercept and $\beta_1$ is the slope coefficient represented by values under harvest strategy.

well as improve water retention and infiltration in the long term.

Potential N mineralization as a proportion of total N decreased with time under all harvest strategies at depths of 0 to 2 and 2 to 4 cm, but was unchanged with time at a depth of 4 to 6 cm (Table 4). The C to N ratio of the mineralizable fraction of organic matter was relatively low at the beginning of the experiment (5.9 at a depth of 0 to 6 cm) and increased with time under all harvest strategies at all soil depths. At the end of 4 years, the mineralizable C to N ratio increased to 16 under the unharvested-forage strategy and to 21 under high grazing pressure. The accumulation of readily available organic substrates with forage-management systems probably led to increased levels of N immobilization to support the growing soil microbial biomass[3], which appears to have reduced net N mineralization. During the first 4 years of bermudagrass management, therefore, actively cycling N in soil appears to be sequestered primarily as PON with little excess mineral N to contribute to the readily mineralizable N pool. It would seem reasonable to predict that upon achieving steady-state levels of all soil N pools at some later

**TABLE 3**
**Rate of Change in Soil N Pools, Based on Linear Regression, During the First 4 Years of Management Summed to a Depth of 6 cm and in Response to Harvest Strategy Averaged across Fertilization Strategies**

| Soil property | Intercept | Harvest strategy UH | LG | HG | H | $LSD_{(P=0.1)}$ |
|---|---|---|---|---|---|---|
| | $g \cdot m^{-2}$ | $g \cdot m^{-2} \cdot year^{-1}$ | | | | |
| Total N | 93.4 | 0.6 | 6.8 | 10.3 | –1.0 | 1.8 |
| Particulate organic N | 14.7 | 3.0 | 7.6 | 9.0 | 2.1 | 1.0 |
| Non-particulate organic N | 75.9 | –1.8 | 0.1 | 2.1 | –2.5 | 1.8 |
| Potential N mineralization in 24 d | 4.86 | –0.31 | –0.20 | –0.17 | –0.42 | 0.16 |

*Note:* UH is Unharvested, LG is low grazing pressure, HG is high grazing pressure, and H is hayed. Linear regression was of the form, (N pool = $\beta_0 + \beta_1 \cdot$ years), where $\beta_0$ is the intercept and $\beta_1$ is the slope coefficient represented by values under harvest strategy.

point in time, the pool of potential N mineralization will then also increase with a C to N ratio more similar to that of total organic C to N. Further research is warranted.

## Fertilization Strategy Impacts

Fertilization strategy had relatively little impact on the dynamics of total N in soil other than a few notable exceptions. At a depth of 0 to 2 cm, the rate of total N accumulation was 0.76 and 0.90 $g \cdot kg^{-1} \cdot year^{-1}$ under inorganic fertilization and averaged 0.57 and 0.70 $g \cdot kg^{-1} \cdot year^{-1}$ under clover-plus-inorganic and broiler litter fertilization under low and high grazing pressures, respectively. On an areal basis to a depth of 6 cm, total N accumulation was 12.7 $g \cdot m^{-2} \cdot year^{-1}$ under inorganic fertilization and averaged 9.1 $g \cdot m^{-2} \cdot year^{-1}$ under clover-plus-inorganic and broiler litter fertilization under high grazing pressure. Fertilization strategy did not consistently affect total N accumulation under the unharvested-forage and haying strategies at any soil depth (data not shown), nor on an areal basis to a depth of 6 cm (Table 5).

Fertilization strategy also affected PON and potential N mineralization at a depth of 0 to 2 cm. Under low grazing pressure only, PON accumulated more with inorganic fertilization (0.47 $g \cdot kg^{-1} \cdot year^{-1}$) than with either clover-plus-inorganic or broiler litter fertilization (0.30 $g \cdot kg^{-1} \cdot year^{-1}$). Potential N mineralization with broiler litter fertilization increased more with time at a depth of 0 to 2 cm than with inorganic-only or clover-plus-inorganic fertilization under high grazing pressure (16 vs. 9 $mg \cdot kg^{-1} \cdot year^{-1}$) and under haying (8 vs. 0 $mg \cdot kg^{-1} \cdot year^{-1}$), but not significantly under the unharvested-forage strategy and low grazing pressure.

At the end of 5 years, inorganic-only fertilization had significantly greater N content in surface residue compared with clover-plus-inorganic and broiler litter fertilization, except when grass was hayed where there was no difference (Table 5). Total soil N content at a depth of 0 to 6 cm was greater with inorganic-only and broiler litter fertilization than with clover-plus-inorganic fertilization under unharvested-forage and haying strategies, but similar under cattle grazing (Table 5). Total soil N content did not differ between inorganic-only and broiler litter fertilization at a depth of 0 to 6 cm, except in April 1998. Overall, it appears that organic and inorganic forms of pasture fertilization were similar in their capacities to sequester soil N.

## Fate of N in Management Systems

Nitrogen fertilizer was applied at a rate of ca. 20 $g N \cdot m^{-2} \cdot year^{-1}$, which would be a typical recommendation for hayed bermuda-grass in the region to achieve 50% of maximum yield and avoid excessive nitrate leaching[14]. Maximum production of 'Coastal' bermudagrass when hayed is likely to occur with application at or in excess of 40 $g N \cdot m^{-2} \cdot year^{-1}$ under normal precipitation for the region[15]. Therefore, N probably limited the hay production and this less-than-optimum fertilization rate resulted in lower soil-profile inorganic N (at a depth of 0 to 1.5 m) during this study compared with initial results (unpublished data).

With an average N application rate of 21.4 $g \cdot m^{-2} \cdot year^{-1}$ across all fertilization strategies, ca. 60% of that N was removed in aboveground forage under haying (estimated using the 5-year average of 0.76 $kg \cdot m^{-2} \cdot year^{-1}$ of dry matter produced and an average N concentration in spring and autumn of 16 $mg N \cdot g^{-1}$ forage). The standing stock of N contained in surface residue at the end of 5 years suggested that 4% of applied N was stored aboveground under hayed management (Table 5). Total soil N at a depth of 0 to 6 cm decreased at a rate of 5% of applied N. Significant losses of N may have occurred via denitrification from soil, volatilization from plants and soil, and runoff. The extent of those processes was not investigated. Changes in soil organic N may have occurred below 6 cm especially in PON sequestered via root turnover. Based on 111 $g N \cdot m^{-2}$ at a depth of 6 to 20 cm at the initiation of this study (unpublished data), an additional 12% of applied N could be accounted for. Therefore, 29% of applied N was unaccounted for under hayed management.

Under unharvested management, the percentage of applied N was 12% in surface residue (Table 5), 3% in total soil N in the surface 6 cm (Table 3), and 20% in total soil N at a depth of 6 to 20 cm. Inorganic N in the 1.5 m profile of soil under unharvested

**TABLE 4**
**Rate of Change in Ratios of Soil N and C Pools, Based on Linear Regression, During the First 4 Years of Management as a Function of Soil Depth and in Response to Harvest Strategy Averaged across Fertilization Strategies**

| | | Harvest strategy | | | | |
|---|---|---|---|---|---|---|
| Soil depth (cm) | Intercept | UH | LG | HG | H | $LSD_{(P=0.1)}$ |
| | $g \cdot kg^{-1}$ | $g \cdot kg^{-1} \cdot year^{-1}$ | | | | |
| | | **Particulate organic N-to-total N ratio** | | | | |
| 0 to 2 | 249 | 32 | 64 | 68 | 19 | 16 |
| 2 to 4 | 102 | 23 | 38 | 43 | 21 | 9 |
| 4 to 6 | 98 | 23 | 35 | 30 | 27 | 10 |
| 0 to 6 | 162 | 30 | 55 | 58 | 24 | 9 |
| | | **Potential N mineralization in 24 d-to-total N ratio** | | | | |
| 0 to 2 | 49.3 | –3.9 | –6.2 | –6.8 | –4.7 | 2.3 |
| 2 to 4 | 64.3 | –7.3 | –6.9 | –7.4 | –8.8 | 2.5 |
| 4 to 6 | 51.5 | –0.0 | –1.1 | –1.4 | 0.5 | 2.5 |
| 0 to 6 | 54.4 | –4.1 | –5.5 | –6.2 | –4.8 | 1.8 |
| | $kg \cdot kg^{-1}$ | $kg \cdot kg^{-1} \cdot year^{-1}$ | | | | |
| | | **Total organic C:N ratio** | | | | |
| 0 to 2 | 14.6 | 0.1 | –0.0 | –0.3 | 0.3 | 0.2 |
| 2 to 4 | 14.4 | 0.6 | 0.5 | 0.4 | 0.9 | 0.3 |
| 4 to 6 | 14.4 | 0.6 | 0.5 | 0.4 | 0.7 | 0.3 |
| 0 to 6 | 14.6 | 0.3 | 0.2 | –0.0 | 0.5 | 0.2 |
| | | **Particulate organic C:N ratio** | | | | |
| 0 to 2 | 24.0 | –0.5 | –1.5 | –2.0 | 0.7 | 0.6 |
| 2 to 4 | 44.4 | –1.8 | –4.4 | –5.6 | –1.4 | 3.0 |
| 4 to 6 | 34.8 | –0.9 | –2.0 | –2.1 | –0.9 | 1.0 |
| 0 to 6 | 28.5 | –0.6 | –2.0 | –2.6 | 0.2 | 0.7 |
| | | **Mineralizable C:N ratio** | | | | |
| 0 to 2 | 10.8 | 3.6 | 4.8 | 6.5 | 6.2 | 2.5 |
| 2 to 4 | 4.4 | 2.7 | 2.6 | 2.4 | 4.0 | 1.4 |
| 4 to 6 | 3.5 | 0.7 | 1.0 | 1.0 | 1.0 | 0.7 |
| 0 to 6 | 5.9 | 2.4 | 3.1 | 3.8 | 3.7 | 1.2 |

*Note:* UH is unharvested, LG is low grazing pressure, HG is high grazing pressure, and H is hayed. Linear regression was of the form, (N pool = $\beta_0 + \beta_1 \cdot$ years), where $\beta_0$ is the intercept and $\beta_1$ is the slope coefficient represented by values under harvest strategy.

management during this study was not different compared with initial results (unpublished data). Therefore, 65% of applied N was unaccounted for.

Under low grazing pressure, the percentage of applied N was 10% in surface residue (Table 5), 32% in total soil N in the surface 6 cm (Table 3), and 31% in total soil N at a depth of 6 to

**TABLE 5**
**Nitrogen Stock in Soil and Residue (g · m$^{-2}$) as Affected by Fertilization Strategy (Inorganic, Clover, and Broiler Llitter) and Harvest Strategy During the First 5 Years of Forage Management**

| | Inorganic | | | | | Clover + inorganic | | | | | Broiler litter | | | | | LSD$_{(P=0.1)}$ | |
|---|---|---|---|---|---|---|---|---|---|---|---|---|---|---|---|---|---|
| Property | UH | LG | HG | H | Mean | UH | LG | HG | H | Mean | UH | LG | HG | H | Mean | All | Means |
| **April 1994** | | | | | | | | | | | | | | | | | |
| Soil (0-6 cm) | 99 | 85 | 86 | 108 | 95 | 81 | 95 | 85 | 82 | 86 | 93 | 81 | 85 | 94 | 89 | 13 | 6 |
| Residue | 8 | 6 | 5 | 6 | 6 | 12 | 6 | 9 | 13 | 10 | 8 | 4 | 5 | 8 | 6 | 3 | 1 |
| Soil + residue | 107 | 90 | 91 | 115 | 101 | 93 | 101 | 93 | 95 | 96 | 101 | 86 | 90 | 102 | 95 | 12 | 6 |
| **April 1995** | | | | | | | | | | | | | | | | | |
| Soil (0-6 cm) | 109 | 100 | 111 | 105 | 106 | 93 | 105 | 104 | 86 | 97 | 103 | 98 | 105 | 110 | 104 | 12 | 6 |
| Residue | 19 | 22 | 12 | 4 | 14 | 22 | 25 | 14 | 6 | 17 | 16 | 21 | 10 | 4 | 13 | 5 | 3 |
| Soil + residue | 128 | 122 | 123 | 109 | 120 | 115 | 130 | 118 | 92 | 114 | 119 | 119 | 115 | 114 | 117 | 10 | 5 |
| **April 1996** | | | | | | | | | | | | | | | | | |
| Soil (0-6 cm) | 98 | 104 | 115 | 101 | 105 | 89 | 111 | 112 | 87 | 100 | 97 | 107 | 114 | 95 | 103 | 10 | 5 |
| Residue | 30 | 22 | 9 | 5 | 17 | 27 | 16 | 12 | 5 | 15 | 17 | 13 | 6 | 3 | 10 | 6 | 3 |
| Soil + residue | 129 | 127 | 124 | 106 | 121 | 116 | 127 | 123 | 92 | 115 | 114 | 120 | 120 | 98 | 113 | 11 | 5 |
| **April 1997** | | | | | | | | | | | | | | | | | |
| Soil (0-6 cm) | 94 | 118 | 119 | 92 | 106 | 84 | 112 | 135 | 87 | 104 | 94 | 110 | 122 | 94 | 105 | 15 | 8 |
| Residue | 30 | 9 | 3 | 4 | 12 | 16 | 8 | 4 | 3 | 8 | 23 | 6 | 2 | 3 | 9 | 6 | 3 |
| Soil + residue | 125 | 127 | 122 | 96 | 118 | 100 | 121 | 139 | 90 | 112 | 117 | 117 | 125 | 97 | 114 | 16 | 8 |
| **April 1998** | | | | | | | | | | | | | | | | | |
| Soil (0-6 cm) | 107 | 128 | 154 | 89 | 120 | 83 | 120 | 123 | 80 | 101 | 101 | 115 | 123 | 89 | 107 | 13 | 7 |
| Residue | 16 | 7 | 4 | 3 | 7 | 7 | 6 | 3 | 4 | 5 | 7 | 4 | 3 | 1 | 4 | 3 | 1 |
| Soil + residue | 122 | 135 | 158 | 92 | 127 | 90 | 126 | 126 | 83 | 106 | 109 | 120 | 126 | 90 | 111 | 14 | 7 |
| **February 1999** | | | | | | | | | | | | | | | | | |
| Soil (0-6 cm) | 152 | 181 | 174 | 137 | 161 | 130 | 183 | 187 | 111 | 153 | 155 | 167 | 183 | 127 | 158 | 17 | 8 |
| Residue | 19 | 14 | 10 | 4 | 12 | 12 | 10 | 8 | 4 | 8 | 9 | 8 | 7 | 4 | 7 | 3 | 1 |
| Soil + residue | 171 | 195 | 185 | 142 | 173 | 142 | 192 | 195 | 115 | 161 | 164 | 175 | 191 | 131 | 165 | 17 | 9 |
| Soil (6–20 cm) | 141 | 147 | 133 | 126 | 137 | 112 | 152 | 140 | 108 | 128 | 143 | 132 | 139 | 137 | 138 | 21 | 10 |

*Note:* UH is unharvested, LG is low grazing pressure, HG is high grazing pressure, and H is hayed.

20 cm. The quantity of N removed with animal gain was 3% of applied N (i.e., based on 1% of live-weight gain as N). Therefore, 24% of applied N was unaccounted for.

Under high grazing pressure, the percentage of applied N was 8% in surface residue (Table 5), 48% in total soil N in the surface 6 cm (Table 3), 23% in total soil N at a depth of 6 to 20 cm, and 3% in animal live-weight gain. Therefore, 18% of applied N was unaccounted for.

In all management systems, a significant portion of total applied N was unaccounted for. Further studies are needed to investigate the quantity of accumulated PON 6 to 20 cm below the soil surface, as well as to quantify gaseous and soluble losses. The surface 6 cm of soil, however, was a large sink for organic N deposited via dung, urine, and dead plant material under grazed systems. Animal grazing systems were more effective at building surface–soil organic N pools that could possibly lead to long-term soil-fertility improvement.

## SUMMARY AND CONCLUSIONS

Grazing of ‘Coastal’ bermudagrass with cattle during five summers was more effective at sequestering total N in the surface

6 cm of soil than unharvested-forage or haying management strategies. Under grazed systems, 81 to 99% of the increase in total N of the surface 6 cm was due to the PON fraction. This fraction represents a passive pool of N derived from above- and belowground plant residues, as well as animal manures, and probably it contributes to both short-term N dynamics and long-term soil fertility. Of the PON sequestered in the surface 6 cm of soil, 75 to 85% occurred in the surface 2 cm of soil. Non-PON increased significantly in all systems at a depth of 0 to 2 cm, but decreased significantly in all systems at a depth of 4 to 6 cm. An increase in potential N mineralization with time occurred only at a depth of 0 to 2 cm under grazed and unharvested systems, but not under hayed management. Whether fertilization was from inorganic or organic forms had relatively little impact on the accumulation of N in soil. The cycling of N from fertilizer sources through forage and cattle led to preferential accumulation of N at the soil surface. Grazing of pastures with cattle may be a viable choice to improve the retention of N within a landscape.

## ACKNOWLEDGMENTS

Thanks are extended to Steven Knapp and David Lovell for their technical expertise in the field and in the laboratory, to Dwight Seman, Fred Hale, Ronald Phillips, and Clara Parker for managing cattle, to Stan Wilkinson for managing forage during early years, and to Latasha Sheats, Terri Cameron, Melissa Walsey, and Patricia Harper for their assistance in the laboratory.

| | | | |
|---|---|---|---|
| Received: | June | 25, | 2001 |
| Revised: | August | 20, | 2001 |
| Accepted: | August | 20, | 2001 |
| Published: | September | 24, | 2001 |
